# Leveraging Rapid Community-Based HIV Testing Campaigns for Non-Communicable Diseases in Rural Uganda

**DOI:** 10.1371/journal.pone.0043400

**Published:** 2012-08-20

**Authors:** Gabriel Chamie, Dalsone Kwarisiima, Tamara D. Clark, Jane Kabami, Vivek Jain, Elvin Geng, Maya L. Petersen, Harsha Thirumurthy, Moses R. Kamya, Diane V. Havlir, Edwin D. Charlebois, the SEARCH Collaboration

**Affiliations:** 1 HIV/AIDS Division, Department of Medicine, San Francisco General Hospital, University of California San Francisco, San Francisco, California, United States of America; 2 Makerere University-University of California San Francisco (MU-UCSF) Research Collaboration, Mbarara, Uganda; 3 Mulago-Mbarara Joint AIDS Program, Kampala and Mbarara, Uganda; 4 School of Public Health, University of California, Berkeley, California, United States of America; 5 Gillings School of Global Public Health, University of North Carolina at Chapel Hill, Chapel Hill, North Carolina, United States of America; 6 Department of Medicine, School of Medicine, Makerere University College of Health Sciences, Kampala, Uganda; 7 Center for AIDS Prevention Studies, Department of Medicine, University of California San Francisco, San Francisco, California, United States of America; University of Ottawa, Canada

## Abstract

**Background:**

The high burden of undiagnosed HIV in sub-Saharan Africa limits treatment and prevention efforts. Community-based HIV testing campaigns can address this challenge and provide an untapped opportunity to identify non-communicable diseases (NCDs). We tested the feasibility and diagnostic yield of integrating NCD and communicable diseases into a rapid HIV testing and referral campaign for all residents of a rural Ugandan parish.

**Methods:**

A five-day, multi-disease campaign, offering diagnostic, preventive, treatment and referral services, was performed in May 2011. Services included point-of-care screening for HIV, malaria, TB, hypertension and diabetes. Finger-prick diagnostics eliminated the need for phlebotomy. HIV-infected adults met clinic staff and peer counselors on-site; those with CD4≤100/µL underwent intensive counseling and rapid referral for antiretroviral therapy (ART). Community participation, case-finding yield, and linkage to care three months post-campaign were analyzed.

**Results:**

Of 6,300 residents, 2,323/3,150 (74%) adults and 2,020/3,150 (69%) children participated. An estimated 95% and 52% of adult female and male residents participated respectively. Adult HIV prevalence was 7.8%, with 46% of HIV-infected adults newly diagnosed. Thirty-nine percent of new HIV diagnoses linked to care. In a pilot subgroup with CD4≤100, 83% linked and started ART within 10 days. Malaria was identified in 10% of children, and hypertension and diabetes in 28% and 3.5% of adults screened, respectively. Sixty-five percent of hypertensives and 23% of diabetics were new diagnoses, of which 43% and 61% linked to care, respectively. Screening identified suspected TB in 87% of HIV-infected and 19% of HIV-uninfected adults; 52% percent of HIV-uninfected TB suspects linked to care.

**Conclusions:**

In an integrated campaign engaging 74% of adult residents, we identified a high burden of undiagnosed HIV, hypertension and diabetes. Improving male attendance and optimizing linkage to care require new approaches. The campaign demonstrates the feasibility of integrating hypertension, diabetes and communicable diseases into HIV initiatives.

## Introduction

There is a high burden of undiagnosed HIV in sub-Saharan Africa, limiting treatment and prevention efforts. [1] Large-scale, community-based HIV testing campaigns can address this challenge and provide an untapped opportunity to identify non-communicable diseases (NCDs). Multiple barriers to diagnosis are shared across diseases, including inaccessible (i.e. expensive or referral lab-based) diagnostics, lack of awareness about the need for testing in persons with minimal or no symptoms of disease, stigma, the high costs to villagers of accessing clinic-based testing (including travel and time away from work), and inadequate or non-existent primary care services. [2], [3], [4] Even after a diagnosis of HIV, TB, malaria, hypertension or diabetes is made, linkage to appropriate clinical care and treatment remains a problem. [5], [6], [7], [8] Through integration of services that can be shared across multiple diseases, such as community mobilization for testing, active case finding, counseling and referral, many of these barriers can be addressed efficiently and simultaneously. With a broad array of point-of-care diagnostic technologies now available, designing a multi-disease field laboratory has become practical.

We sought to test the feasibility and diagnostic yield of integrating NCD and other communicable disease services into a rapid, high-throughput, community-based HIV testing and referral campaign for all residents of a rural Ugandan parish, and to determine rates and predictors of post-campaign linkage to care by disease.

## Methods

### Campaign Design

The community health campaign was designed based on collaboration between Makerere University – University of California, San Francisco (MU-UCSF) investigators and the Mulago-Mbarara Joint AIDS Program (MJAP) in Uganda, with Ministry of Health (MOH) support. The campaign was implemented in Kakyerere parish, Mbarara District, a rural community in southwestern Uganda and designed to deliver free, high-throughput (>1,000 residents/day), multi-disease services (Table 1) to all parish residents over five days in May 2011. Staff members were hired locally for a two-week period: one week for training on campaign procedures (culminating in a one-day dress rehearsal of the campaign procedures), and one week for the campaign. All staff had prior training and work experience to match their role within the campaign. The pre- and post-test counselors had training and counseling experience from HIV clinics in Mbarara municipality. The laboratory workers were hired from local public and private clinical laboratories and were required to have, at a minimum, basic skills in finger-prick based diagnostics including rapid HIV-testing. Staff with interview questionnaire experience and computer literacy performed consent procedures and computer-based questionnaires, including symptom screening. All staff members were fluent in the local language, Runyankole.

**Table 1:** High Throughput Kakyerere Parish Community Health Campaign Services.

Service Category	Service	Population
Diagnosis	HIV antibody testing^a^	All participants
	Point-of-care CD4+ testing^b^	HIV+ participants
	TB symptom screening^c^	All participants
	Near point-of-care TB sputum screening^d^	HIV+ participants
	Malaria screening^e^	Participants reporting fever in the past 24 hours^m^
	Diabetes screening^f^	Adult participants
	Hypertension screening^g^	Adult participants
Prevention	Pre- and Post-test counseling for HIV, suspected TB, malaria, hypertension, and diabetes	All participants
	TMP/SMX –30 day supply^h^	HIV+ participants
	Male condoms	Adult participants
	Vitamin A 200,000 IU^i^	Children: 6 months –5 years
	Insecticide-treated bed nets^j^	All households
Treatment	Artemether-Lumefantrine^k^	Malaria+ participants
	Mebendazole 500 mg^l^	Children: 1–5 years
Referral	Routine scheduled appointments to a local health centre	Participants with HIV, Suspected TB, Diabetes, or Hypertension
	Rapid appointments (<2 weeks) with expedited ART initiation	HIV+ and CD4≤100 cells/µL

a Serial testing algorithm, Uganda National Policy Guidelines for HIV Voluntary Testing and Counseling. [9] HIV rapid test kits: Determine HIV-1/HIV-2 (Abbot); HIV-1/2 Stat-Pak assay; and Uni-Gold HIV Rapid test.

b PIMA CD4 (Inverness). Indeterminate CD4+ cell count results were repeated once. Participants with two indeterminate CD4 results were counseled to undergo clinic-based CD4 cell count testing.

c HIV+: WHO symptom screening algorithm (current cough, fever, sweats or weight loss) [28]; HIV-: cough >2 weeks.

d Xpert MTB/RIF Assay (Cepheid) and direct fluorescent microscopy. Indeterminate Xpert assay results were repeated one time.

e Paracheck Pf (P. falciparum) Rapid Test (Orchid Biomedical Systems).

f Random blood glucose ≥11.1 mmol/L [32], HemoCue (Quest Diagnostics).

g Systolic blood pressure ≥140 mmHg or diastolic blood pressure ≥90 mmHg. [33] All blood pressure measures were performed using automated, electronic pressure cuffs, with small, normal and large cuff sizes available. Blood pressure measures were performed one time; any participant with a positive screen underwent two repeat measurements, including one manual blood pressure cuff measurement.

h WHO guidelines on co-trimoxazole prophylaxis for HIV-related infections [34].

i WHO guidelines on vitamin A supplementation [35].

j Distributed to all parish households after completion of the campaign.

k For treating uncomplicated malaria. Severe malaria cases were provided transport to the nearest government hospital.

l WHO/Unicef guidelines, 2004 [36].

m Cases of fever with hypotension were provided immediate transport to the nearest government hospital. HIV-uninfected participants with non-malarial fever were counseled to seek further evaluation at the nearest government clinic.

### Campaign Setting and Pre-existing Health Services

**Figure 1:**
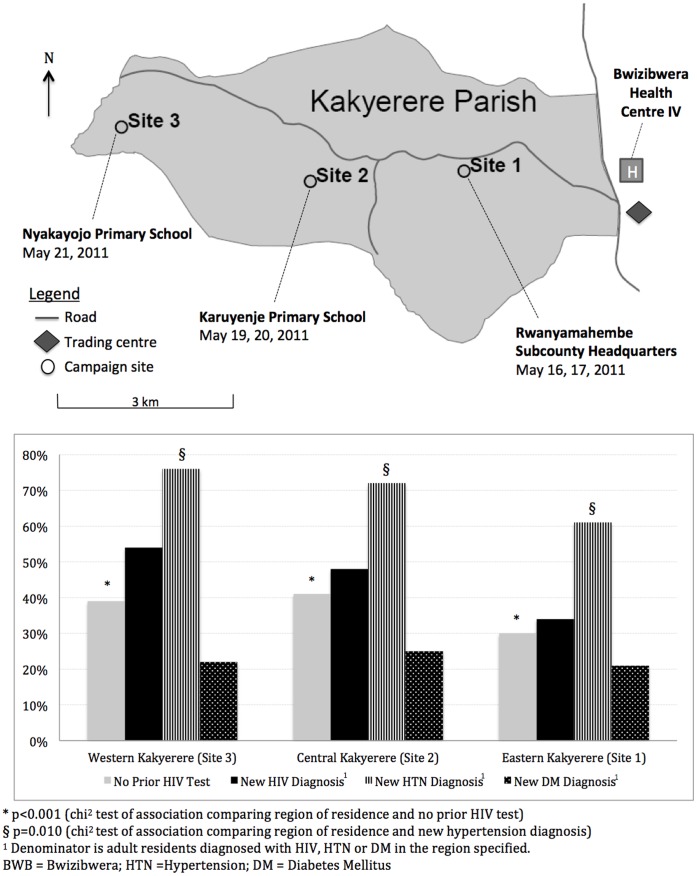
Map of Kakyerere Parish. Location of Campaign Sites and the Relationship Between Prevalence of Undiagnosed Disease among Adult Parish Residents and Increasing Distance from the Local Health Centre.

Kakyerere Parish in Mbarara District is a rural community composed of nine villages and 6,300 residents in southwestern Uganda. The parish’s eastern boundary ends at Bwizibwera trading centre (see Figure 1: map), a central market and public gathering place that is the site of Bwizibwera Health Centre IV (BWB HCIV), the primary government clinic for Kakyerere residents. The western-most village in Kakyerere is nine kilometres from BWB HCIV (see map). BWB HCIV offers diagnostic and treatment services for HIV (including antiretroviral therapy), active TB and pregnancy, as well as acute medical (e.g. malaria) and surgical services. Though diagnostic services for hypertension and diabetes are available, treatment of hypertension and diabetes is not offered at BWB HCIV. The nearest government clinic offering pharmacologic treatment for hypertension and diabetes is located approximately 20 km from BWB HCIV at the Mbarara University of Science and Technology (MUST) Hospital in Mbarara Municipality.

### Community Mobilization

All local village administrative councilors from Kakyerere Parish were engaged in planning the campaign to ensure that services addressed parish needs. They designed and executed community mobilization activities one month before the campaign, including church and mosque announcements, distribution of posters, and radio announcements. To maximize participation and reduce transport costs, campaign activities took place at three well-known locations across the parish (Figure 1).

### Laboratory Testing

Laboratory testing occurred in the field and in a central laboratory in Mbarara municipality. The field laboratory offered rapid point-of-care HIV antibody testing, diabetes screening and malaria screening with a single finger-prick (Table 1). Blood glucose measures were random, as neither fasting nor two-hour measures were compatible with the high-throughput design. Point-of-care (<20 minute) CD4+ cell count testing (PIMA, Inverness Medical) was performed in all HIV-infected participants with an additional finger-prick.

At the central laboratory, sputum microscopy and rapid PCR-based, TB sputum testing (Xpert MTB/RIF Assay, Cepheid) were performed. Sputum was collected from all consenting HIV-infected adults, with induction using nebulized hypertonic saline for participants unable to spontaneously produce sputum. All sputum samples were brought to the central laboratory within three hours of collection for evaluation. Samples that could not be processed immediately were stored at 4°C.

### Disease Screening

Definitions for a positive screening test for HIV, TB, malaria, diabetes and hypertension are shown in Table 1.

### Linkage to Care

Successful linkage to care was defined as attending at least one appointment for a specific diagnosis in the three months after the campaign.

#### Routine linkage

Participants with HIV, suspected TB, hypertension or diabetes received post-test counseling and a stipend to cover travel to clinic. HIV-infected participants met with clinical staff and HIV-infected peer counselors on-site. HIV-infected participants and HIV-uninfected TB suspects were scheduled appointments at a local clinic (Bwizibwera Health Centre) and offered enrollment in a linkage to care sub-study in which they provided identifying information and agreed to be located and interviewed should they not link to care. Appointment scheduling took CD4 cell count into consideration, with earlier appointments for participants with lower CD4 counts. Participants with diabetes and hypertension were referred to a government diabetes and hypertension clinic in Mbarara Municipality that provides oral hypoglycemic and anti-hypertensive medications.

#### Enhanced linkage

HIV-infected participants with CD4 count ≤100 cells/µL were offered enrollment in an enhanced strategy of rapid referral (<2 weeks post-campaign), expedited pre- antiretroviral therapy (ART) counseling and ART initiation at first clinic visit. The objective of the enhanced linkage pilot was to determine if a government clinic that requires ART eligible patients to attend three adherence counseling visits and to bring a friend/relative as evidence of adherence support prior to ART, could change its practices for a high-risk population. The strategy of ART start at first clinic visit was piloted among participants with CD4<100 so as to focus on those with the most advanced HIV disease who are at highest risk of morbidity and mortality within a short time frame.

### Statistical Methods

Prevalence of each disease was calculated as a proportion with the number of participants tested for each disease as the denominator. Proportions were compared using the Pearson χ^2^ test, means by the Student’s t-test, and medians by the Wilcoxon rank sum test. Multivariable logistic regression was used to evaluate risk factors for hypertension and diabetes, and predictors of linking to care among newly diagnosed HIV-infected adults and HIV-uninfected TB suspects. Independent variables for each logistic regression analysis are described below and included age, gender, and variables that were significantly associated with the outcome of interest (p<0.1) on univariate analysis. Cost data are reported based on actual expenditures incurred for each component of the campaign (including equipment, salary, and facility rental costs), and presented as costs per participant. We performed all statistical analyses using Stata/SE version 12.

### Ethical Statement

Campaign activities were anonymous. As written consent requires recording of participants’ names and would have resulted in confidential but not anonymous campaign participation, verbal consent was obtained for participation. Children from 13–17 years could provide verbal consent, consistent with Uganda MOH policy. [9] Children <13 years could not participate without a parent/guardian present. The linkage to care sub-study required written consent, as identifying information was collected. The Makerere University School of Medicine Research and Ethics Committee, the Ugandan National Council on Science and Technology, and the UCSF Committee on Human Research approved the study.

## Results

### Community Participation

Over five days in one week, 4,343 people attended the campaign: 2,323 adults and 2,020 children (<18 years). Absolute numbers of participants were highest in the central region of the parish, with 984 people receiving services in one day. Participants spent a median of 95 minutes (IQR: 71–129) at the campaign. HIV-infected participants spent more time at the campaign (171 minutes [IQR: 136–216]) than HIV-uninfected participants (93 minutes [IQR: 70–125]).

Based on population projections from the 2002 Ugandan Census that use an annual estimated growth rate of 2.16%, there were an estimated 6,300 Kakyerere parish residents in 2011, with 50% of the population ≥18 years. [10] Therefore, an estimated 2,323/3,150 (74%) of adult residents, and 2,020/3,150 (69%) of child residents, participated in the campaign. Of an estimated 1,600 female and 1,550 male adult residents, 1,523 (95%) women and 800 (52%) men attended. Among child residents, participation rates were similar between girls and boys (1,066 girls and 954 boys attended: 67% vs. 62%, respectively). Demographic characteristics of campaign participants are shown in Table 2.

**Table 2:** Demographic Characteristics of Kakyerere Parish Community Health Campaign Participants.

All Participants	N	%
Age (median [IQR]), in years	19 [8–38]	
Adults (≥18 years)	2,323	53
Children (<18 years)	2,020	47
Age Deciles (years)		
0–9	1,198	27.6
10–19	974	22.4
20–29	636	14.6
30–39	525	12.1
40–49	424	9.8
50–59	247	5.7
60–69	177	4.1
>70	153	3.5
Adult, age unknown	9	0.2
Total	4,343	100
**Adult Participants**	**N = 2,323**	**%**
Female	1,523	66
Male	800	34
Marital Status		
Single	315	14
Married	1,530	66
Divorced/Separated	172	7
Widowed	306	13
Occupation		
Farmer	1,643	71
Shopkeeper	130	6
Student	111	5
Transport	38	2
Market Vendor	47	2
Other	354	15
Education Attainment		
No schooling	569	24
Completed primary school	474	20
Completed secondary school	94	4

### Active Case Finding

The burden of HIV, suspected TB, malaria, hypertension and diabetes among campaign participants are shown in Table 3.

**Table 3:** Communicable and Non-Communicable Disease Burden and Linkage to Care Rates Among Campaign Participants.

	HIV	Malaria	Suspected TB^1^			Hypertension	Diabetes Mellitus
Prevalence			HIV+	HIV-			
Adults (≥18 years)	179/2,282 (7.8%)	16/633 (2.5%)	155/179 (87%)	392/2,103 (19%)		645/2,278 (28.3%)	80/2,283 (3.5%)
15–49 years	163/2,000 (8.1%)	16/542 (3.0%)	141/163 (87%)	310/1,846 (17%)		388/2009 (19.3%)	32/2009 (1.6%)
Children (18 mos - 17 yrs)	10/1,826 (0.5%)	45/534 (8.4%)^2^	10/10 (100%)^2^	455/1,816 (25%)^2^		N/A	N/A
**Adult CHC Participants (N = 2,323)**	**N = 179**	**N = 16**	**N = 155**	**N = 392**		**N = 645**	**N = 80**
New Diagnosis	82/179 **(46%)**	16/16 **(100%)**	N/A	N/A		422/645 **(65%)**	18/80 **(23%)**
Linked to care post-campaign	Routine: 20/58 **(34%)** Enhanced: 5/6 **(83%)** Overall: 25/64 **(39%)** 3	N/A (Treated on-site)	71/122 **(58%)** 3	130/252 **(52%)** 3		107/249 **(43%)** 4	11/18 **(61%)** 4
Prior Diagnosis	97/179 **(54%)**	N/A	N/A		N/A	223/645 **(35%)**	62/80 **(77%)**
In clinic-based care pre-campaign	96/97 (99%)	N/A	83/155 (54%)		N/A	N/A	N/A
Receiving treatment pre-campaign	ART: 66/97 (68%)TMP/SMX: 45/97 (46%)	N/A	2/155 (1%)^5^		15/392 (4%)^5^	85/223 (38%)	38/62 (61%)
% Not on ART of those in HIV care & CD4≤350	17/44 (39%)	N/A	12/34 (35%)		N/A	N/A	N/A
% Not on ART of those in HIV care & CD4≤250	8/24 (33%)	N/A	4/16 (25%)		N/A	N/A	N/A

1 TB suspect defined in HIV-uninfected as cough >2 weeks, and in HIV-infected as current cough or fever, or any recent night sweats or weight loss.

2 Symptoms reported by parent/guardian if child <13.

3 Denominator is adults newly diagnosed with HIV or suspected TB who consented to participate in the linkage to care sub-study.

4 Denominator is adults newly diagnosed with hypertension or diabetes who scheduled appointments at the government-run hypertension/diabetes clinic.

5 Participant-reported TB treatment.

N/A = not applicable or not asked.

#### HIV

Among 2,323 adult participants, 2,282 (98%) underwent HIV antibody testing, 12 (0.5%) declined and 29 (1.2%) were not tested; 179/2,282 (7.8%) tested positive. Of 1,826 participants aged 18 months –17 years, 10 (0.5%) tested HIV antibody positive. Adult HIV prevalence was higher in women than men (9.2 vs. 5.2%, respectively; p = 0.001). 802 (35%) adults reported never previously testing for HIV, and adults living in the central and western parish regions reported significantly higher rates of never HIV testing compared to adults living in the more urban eastern region (with a trading and health centre; see Figure 1).

Among HIV-infected participants, 82/179 (46%) adults and 7/10 children were newly diagnosed with HIV at the campaign. The proportion of new HIV diagnoses was greater among HIV-infected adults living in the western (54%) and central (48%) regions, compared to those in the eastern region (34%), although this difference was not statistically significant (p = 0.11).

CD4 cell counts were obtained in 167 (93%) HIV-infected adults and the median CD4 count was 415 (IQR: 281–568) cells/µL. Among adults not on ART, new HIV diagnoses had a higher median CD4 count (449 cells/µL [IQR: 281–592]; n = 77) than prior diagnoses (345 cells/µL [IQR: 279–521; n = 28), though the difference was not statistically significant (p = 0.28).

#### Suspected TB

Among 179 HIV-infected adults, 155 (87%) reported current cough, fever in the past 24 hours, any recent weight loss or night sweats. 101 of 155 (65%) TB suspects agreed to field sputum collection and produced sputum; all 101 tested AFB smear negative, and 100 tested negative by the Xpert MTB/RIF assay (with one indeterminate result).

Of HIV-uninfected adults, 392/2,103 (19%) reported a current cough of >2 weeks duration. All 392 TB suspects received counseling and referral with a transport stipend to the local health centre for further evaluation.

#### Malaria

Of 1,331 children ≤10 years old, 531 (40%) had a subjective fever reported by a parent or guardian over the preceding 24 hours; 386 (73%) underwent malaria rapid diagnostic testing (RDT), and 38 (10%) tested RDT positive. In participants >10 years, 1,188/3,012 (39%) reported fever, 781/1,188 (66%) underwent malaria RDT, and 23/781 (3%) tested positive. Due to a RDT shortage on day two, 145 (27%) children ≤10 years and 407 (34%) participants >10 years with fever were not tested.

#### Hypertension

Of 2,323 adult participants, 2,278 (98%) underwent hypertension screening and 645/2,278 (28%) had a systolic blood pressure ≥140 mmHg, a diastolic blood pressure ≥90 mmHg or reported a prior diagnosis of hypertension. 422 (65%) hypertensive adults reported no prior diagnosis of hypertension. The prevalence of hypertension was similar among women (29%) and men (27%; p = 0.4), and did not vary significantly by region of residence (eastern (29%) vs. central (25%) vs. western (30%); p = 0.19). Adults with hypertension tended to be older (median age: 46 years [IQR: 35–60]) than those without hypertension (32 years [24–45]; p<0.001), and have lower education attainment (any education: 66% vs. 79%, respectively; p<0.001). In a multivariate logistic regression model that included age, sex, marital status and education attainment, only age remained significantly associated with hypertension. The proportion of new hypertensive diagnoses was significantly greater in male (162/213 [76%]) than female participants (260/432 [60%], p<0.001) and in residents of the central and western parish regions compared to the eastern region (Figure 1).

#### Diabetes mellitus

Diabetes screening was performed in 2,283 (98%) adult participants, and 80 (3.5%) had a blood glucose ≥11.1 mmol/L or reported a prior diagnosis of diabetes; 18/80 (23%) reported no prior diabetes diagnosis. Adults with diabetes tended to be older (median age: 52 years [IQR: 37–65]) than those without diabetes (35 years [25–48]; p<0.001), to own more land (mean acres owned: 6.9 vs. 4.1; respectively; p = 0.002), to have higher education attainment (completed primary school: 7.6% vs. 3.3%, respectively; p = 0.02), and to live in the eastern (4.4%) vs. the central (2.2%) and western (2.7%) regions (p = 0.06). In a multivariate logistic regression model controlling for age, gender, education, region of residence, and acres owned, older age (OR = 1.72 for each decade increase, [95% CI: 1.45–2.04], p<0.001), living in the eastern vs. central or western regions (OR = 2.02 [95% CI: 1.17–3.50], p = 0.01) and having any compared to no education (OR = 2.72 [95% CI: 1.10–4.70], p = 0.03) remained positively associated with diabetes.

### Linkage to Care

Of 82 adults with newly diagnosed HIV, six had CD4≤100 cells/µL, 71 had CD4>100, and five had indeterminate CD4 counts. Of the 82 adults, 64 (78%) enrolled in the linkage to care study: 58 in the routine strategy (including one with CD4 = 85 who declined enhanced referral) and six in the enhanced strategy (five with CD4≤100 and one pregnant woman in her third trimester with CD4 = 278). Of 18 adults with newly diagnosed HIV who did not enroll in the linkage study, 13 were missed due to implementation errors in the first two days of the campaign. Two participants with CD4 counts of 106 and 4 cells/µL who reported a prior HIV diagnosis but were not on ART also enrolled in the enhanced strategy. Participants referred with the enhanced strategy had a median CD4 count of 64 cells/µL (IQR: 22–97) vs. 441 cells/µL (IQR: 299–572) among routine referrals.

After routine referral, 20/58 (34%) adults with newly-diagnosed HIV linked to HIV care within three months. After enhanced referral, 6/8 (75%) adults linked to HIV care, including 5/6 (83%) new diagnoses; all six participants linked within ten (median 2.5) days of the campaign, screened negative for active TB by sputum evaluation and/or clinician assessment, started ART at the first clinic visit, and reported medication adherence four weeks after starting ART. The odds of linking to care among new HIV diagnoses decreased significantly with increased point-of-care (POC) CD4 count (unadjusted OR: 0.80 for every 100 cell increase in CD4 cell count, 95% CI: 0.64–1.00; p = 0.048); this association remained significant after adjusting for age, sex, and distance from clinic (adjusted OR: for every 100 cell increase in CD4 count, 95% CI: ). There were no statistically significant differences in age, sex, distance of residence from clinic, marital status or symptoms between adults who did and did not link. However, there was a non-significant trend towards increased linkage in adults living closer to the local clinic (eastern region: 8/16 (50%); central region: 14/32 (38%); western region: 2/10 (20%), linked to care; p = 0.30; among 58 adults with newly-diagnosed HIV who provided location of residence data).

We attempted to track and interview 35 of the 39 adults with newly diagnosed HIV who did not link to care: 20 could not be located despite multiple attempts to find their homes, eight had moved from Kakyerere parish (primarily to find work), five provided reasons for not linking that suggested fear of stigma at the health centre, one reported insufficient transport funds, and one did not believe her HIV diagnosis was correct.

Among HIV-uninfected adult TB suspects, 252/392 (64%) enrolled in the linkage to care sub-study. 130/252 (52%) linked to care; all 130 produced sputum and tested AFB smear negative. The unadjusted odds of linking to care for TB evaluation among HIV-uninfected adults was higher with increasing age (OR: 1.3 for every 10 years of age, 95% CI: 1.1–1.5; p<0.001), in women vs. men (OR: 1.8, 95% CI: 1.1–3.0; p = 0.028), in married vs. single adults (OR: 6.2, 95% CI: 2.0–18.7; p = 0.001), in persons reporting night sweats vs. no night sweats (OR: 1.8, 95% CI: 1.1–3.1, p = 0.027) and in the eastern vs. western region (OR: 2.6, 95% CI: 1.2–5.5, p = 0.016). In a multivariate model including these variables, the odds of linking to care remained higher with increasing age (OR: 1.3 for every 10 years, 95% CI: 1.1–1.7; p = 0.009), night sweats (OR: 2.0, 95% CI: 1.0–3.9; p = 0.047), and residence in the eastern vs. western region (OR: 3.0, 95% CI: 1.3–6.8; p = 0.009).

Among 422 adults with newly diagnosed hypertension, 249 (59%) accepted referral appointments and 107 (43%) linked to care within three months. All 18 adults with newly diagnosed diabetes accepted referral appointments and 11 (61%) linked within three months.

### Campaign Costs

The cost of providing the full multi-disease campaign and evaluation was $37.82 (2011 US dollars) per participant. The cost of providing HIV counseling and testing (HCT) alone (including costs of the high throughput community campaign infrastructure) was $8.27 per participant. The cost of combined HIV services at the campaign, including HCT, POC CD4 ($16.42/participant), trimethoprim-sulfamethoxazole ($0.02/participant), and linkage to care services (including travel stipends) was $26.69 per participant. The incremental cost of adding components on top of the combined HIV services was $2.41 for diabetes and hypertension screening, $4.58 for TB screening (including rapid, PCR-based TB testing), and $2.11 for malaria rapid diagnostic testing, treatment and insecticide-treated bednets.

## Discussion

Our campaign demonstrates the feasibility of integrating communicable and non-communicable disease (NCD) screening into a community-wide HIV testing drive in rural Africa. The campaign reached 74% of adults in a community of 6,300 people rapidly (in five days) with efficient, high-throughput (95 minutes/person) use of point-of-care diagnostics. We identified a high burden of undiagnosed diseases using active case finding, even though multi-disease diagnostic capacity existed prior to the campaign at a nearby, local clinic. Active screening resulted in detection of HIV cases at high CD4 counts (median: 415), well above country guidelines for ART initiation. [11] Improving male attendance and optimizing linkage to care rates require new approaches.

In contrast to health campaigns that focus efforts on a single disease or demographic or that require intensive resources for home-based testing, we focused on overcoming barriers to diagnosis and referral that are shared by multiple diseases in resource-limited settings. [12] These barriers include community participation, field laboratory infrastructure, access to trained counselors, and referral services. Furthermore, by combining services in a public setting, HIV testing is normalized as a routine part of health care, thus reducing stigma. Importantly the campaign represents a step forward in the rapid scale-up of integrated communicable and non-communicable disease services in a rural African setting, and builds on the prior successes of high-throughput HIV-testing campaigns. [13]

The cost estimate for the full multi-disease campaign ($37.82/participant) reflects start-up and research evaluation costs; these costs will decrease with more widespread implementation, increased number of people tested and streamlining. Our initial cost estimates are slightly less than published costs for an HIV counseling and testing (HCT) campaign with water filter and bednet distribution in rural Kenya ($40.66/person), [14] and the cost of HCT services alone using our community campaign approach is comparable to estimates of door-to-door HCT. [12] Community-based testing offers additional advantages by serving as a platform for multi-disease delivery. The relatively low cost of adding NCD screening to community-based HIV testing campaigns ($2.41/person) highlight this latter advantage. This study does not address the cost-effectiveness of adding NCD screening, but the relatively low cost of $2.41/person makes it likely to be cost-effective. Furthermore, the early HIV diagnoses made possible by the campaign (i.e. a median CD4 of 499 cells/µL among new HIV diagnoses), could result in substantial benefit through reductions in morbidity and mortality, as well as HIV transmission, particularly if coupled with earlier ART initiation.

We identified a substantial burden of hypertension and diabetes in this population; further evidence that NCDs are a global health threat rather than a problem of high-income countries alone. [15] Our estimates of hypertension and diabetes prevalence are within the range of published estimates for east Africa. [16], [17], [18]_ENREF_14 NCD burden will increase with urbanization and ageing of populations worldwide, [19] and our results reflect this; increasing age was associated with hypertension and diabetes, and higher education attainment was associated with diabetes. At a recent high-level United Nations meeting to address the growing global NCD crisis, priorities for action focused on prevention (e.g. tobacco control, salt reduction), primarily at the national level. [20] These priorities are important, but do little for individuals already living with NCDs._ENREF_23 We show that HIV testing and referral can be leveraged in rural Africa to find and engage patients with undiagnosed NCDs, though capacity for NCD treatment remains limited. The substantial investment in HIV infrastructure in sub-Saharan Africa presents an important opportunity and an immediate way forward for delivering NCD care in resource-limited settings.

Linkage to care after referral was insufficient and undermines the benefits of early diagnosis. Low rates of linkage to care after an HIV diagnosis and loss to follow-up are common in Africa, due in part to the large number of steps between diagnosis and ART initiation, including send-out CD4 count testing. [7] Point-of-care (POC) CD4 count testing can reduce time to ART start in patients enrolled in HIV care and those diagnosed with HIV in a clinical setting. [21], [22] We used POC CD4 with community-based HIV testing to triage referrals based on CD4 count and to accelerate ART start (<10 days post-diagnosis) in a small, pilot group of high-risk patients (CD4<100). Future interventions are needed to optimize linkage to care for HIV-infected persons living in remote areas, including de-centralized, streamlined care and re-engagement for those who have dropped out of care.

Our estimation of linkage to care has several limitations. First, we measured linkage to care at a single clinic, BWB HCIV, and defined successful engagement in care as an initial visit within three months of diagnosis. Residents may have sought HIV, hypertension or diabetes care elsewhere, or engaged in care later than three months after diagnosis, resulting in underestimation of linkage. Second, linkage to an initial clinic appointment may not result in sustained engagement in care, and assessment of the long-term health effects of early HIV or NCD diagnosis is beyond the scope of this analysis. However, even without long-term outcome data, active community-based HIV and NCD diagnoses and linkage to a first clinic visit remain steps required for successful engagement in care and are likely to have a sustained impact on HIV and NCD control in this community. [23], [24], [25]

Increasing adult male participation and optimizing TB case finding are two challenges that future campaigns will address. A high proportion of adult parish residents participated, but men attended at far lower levels than women, consistent with prior studies of HIV testing. [13], [26] Male reticence to participate in health care, perceptions of illness as a sign of weakness and vulnerability, and extended work hours that conflicted with the campaign’s schedule may account for lower male participation. [26], [27] More research is needed to clarify obstacles and evaluate incentives to increase male participation. Future campaigns will pilot a day exclusively for men to undergo testing, syphilis screening, and hours to accommodate work schedules.

With WHO guideline-based TB symptom screening designed to exclude active TB, we successfully identified 13% of HIV-infected adults for safe initiation of isoniazid preventive therapy (IPT). [28] However, the high prevalence of TB symptoms in this rural setting (19% of HIV-uninfected adults) resulted in large numbers of TB suspects requiring further lab-based evaluation. The Xpert MTB/RIF assay was used to evaluate HIV-infected TB suspects, but no Xpert-positive TB cases were identified, likely due to the number screened (100 suspects); the expected prevalence of newly diagnosed TB among HIV-infected persons in population-based surveys is <1% in resource-limited settings. [29] Cases may have also been missed as the sensitivity of the Xpert assay from a single sample in smear negative TB is 43–72%, and lower in HIV-infected than uninfected persons. [30], [31] Further investigation is needed into TB diagnostic algorithms following positive symptom screening and improving diagnostics for settings where MTB culture is not available. In addition, with the low rate of linkage to care after positive symptom screening and the low yield of active TB in HIV-uninfected campaign participants, novel active TB case finding strategies are needed that account for local HIV and TB epidemiology.

At this juncture, given the substantial investment in HIV infrastructure in sub-Saharan Africa to date, there is a widespread opportunity to leverage HIV infrastructure for integrated disease management. The numerous lessons learned from HIV prevention and treatment implementation can and should be a basis for management of chronic diseases, including the growing NCD burden in Africa. Our campaign represents a community-wide effort to jump-start the process of integration.
